# Postoperative abdominal complications after cardiopulmonary bypass

**DOI:** 10.1186/1749-8090-7-108

**Published:** 2012-10-09

**Authors:** Guohua Dong, Canhui Liu, Biao Xu, Hua Jing, Demin Li, Haiwei Wu

**Affiliations:** 1 Department of Cardiovascular and Thoracic Surgery, Jinling Hospital, Clinical Medicine School of Nanjing University, 305 East Zhongshan Road, Nanjing, 210002, Jiangsu, China

**Keywords:** Abdominal complications, Cardiopulmonary bypass, Cardiovascular surgery

## Abstract

**Background:**

To summarize the diagnostic and therapeutic experiences on the patients who suffered abdominal complications after cardiovascular surgery with cardiopulmonary bypass(CPB).

**Methods:**

A total of 2349 consecutive patients submitted to cardiovascular surgery with CPB in our hospital from Jan 2004 to Dec 2010 were involved. The clinical data of any abdominal complication, including its incidence, characters, relative risks, diagnostic measures, medical or surgical management and mortality, was retrospectively analyzed.

**Results:**

Of all the patients, 33(1.4%) developed abdominal complications postoperatively, including 11(33.3%) cases of paralytic ileus, 9(27.3%) of gastrointestinal haemorrhage, 2(6.1%) of gastroduodenal ulcer perforation, 2(6.1%) of acute calculus cholecystitis, 3(9.1%) of acute acalculus cholecystitis, 4(12.1%) of hepatic dysfunction and 2(6.1%) of ischemia bowel diseases. Of the 33 patients, 26 (78.8%) accepted medical treatment and 7 (21.2%) underwent subsequent surgical intervention. There were 5(15.2%) deaths in this series, which was significantly higher than the overall mortality (2.7%). Positive history of peptic ulcer, advanced ages, bad heart function, preoperative IABP support, prolonged CPB time, low cardiac output and prolonged mechanical ventilation are the risk factors of abdominal complications.

**Conclusions:**

Abdominal complications after cardiovascular surgery with CPB have a low incidence but a higher mortality. Early detection and prompt appropriate intervention are essential for the outcome of the patients.

## Background

Abdominal complications following cardiopulmonary bypass (CPB) have been reported since the early days of cardiac surgery. Despite improvements in surgical techniques and perfusion technologies, the incidence and mortality have not decreased during the last two decades. It remains a significant problem in the clinical practices. Early diagnosis and aggressive treatment is the key to successfully reduce mortality [1]. Unfortunately, the clinical assessment is often difficult because these patients may be sedated, mechanically ventilated, partially unresponsive, and that the usual symptoms and signs may be masked by severe cardiac and pulmonary conditions. In this study, we retrospectively analyzed a total of 2349 patients who underwent cardiovascular surgery with CPB at our institution from Jan 2004 to Dec 2010, and identified the clinical characteristics of the patients who suffered from postoperative abdominal complications, aiming to find ways to improve the clinical outcome.

## Methods

The consecutive patients submitted to cardiovascular surgery with CPB in our hospital from Jan 2004 to Dec 2010 were involved. Every operation involved the use of moderately hypothermic (28-32°C) CPB perfusion. Myocardial preservation was achieved with cold (4°C) crystalloid cardioplegia and warm blood using both anterograde and retrograde techniques, which was supplemented with topical hypothermia in pericardial cavity. During cardiopulmonary bypass, haematocrit was kept minimally at 22-25%.

All patients had a nasogastric tube placed during surgery and this remained in position for at least 6 h after operation. They also received prophylactic intravenous antibiotics, and conventional liquid or blood infusion if necessary. In patients with postoperative low cardiac output (LCO), inotropic pharmacological support was required, with or without intra-aortic balloon pump (IABP) insertion. The usual inotropic pharmacological support was dopamine/duobafendin or amrinon/milrinone, combined with additional vasoconstrictor, such as adrenalin or phenylephrine if necessary. Our routine prophylaxis antacid against ulcers was done with ranitidine on the intensive care unit (ICU). A proton channel blocker (omeprazole) was only used in patients with a history of ulcers. Anticoagulant therapy was applied in all cases that had undergone valve(s) replacement and/or vascular prosthesis implantation.

Abdominal complications were classified as following, paralytic ileus, gastrointestinal haemorrhage, gastroduodenal perforation, acute calculus cholecystitis, acute acalculus cholecystitis, hepatic dysfunction and ichemic bowel disease. Patients with transient intestinal ileus, mild hepatic dysfunction, or asymptomatic jaundice were not included. Other rare complications such as acute pancreatitis and pseudomembranous colitis were not encountered in our series.

The clinical data of any abdominal complication, including its incidence, characters, relative risks, diagnostic measures, medical or surgical management and mortality, was retrospectively analyzed. Statistical analysis was performed using non-paired Student's *t*-test and *χ*^2^ analysis. Data were considered significant at the P < 0.05 level.

## Results

A total of 2349 consecutive patients with CPB were involved. The brief operative characteristics of total patients were summarized in Table 1. A history of gastrointestinal ulcer diseases was present in 9 patients, 59 patients were classified in NYHA class IV, and 6 patients needed preoperative IABP support. Abdominal complications occurred postoperatively in 33 with an incidence of 1.4%. This population included 19 men (57.6%) and 14 women (42.2%) and their mean age was 43.8 years (4-86 years).

**Table 1:** Characteristics of total patients

**Variable**	**Vavle**
Age (years)	52.1 ± 33.7
Gender(male/female)	1325/1024
Preexisting illness	
Hypertension	175
Diabetes	126
Previous MI	37
Heart failure	83
Operative priority	
Elective	2148
Non-elective	201
Type of surgery	
CABG	215
Valve replacement/Valvuloplasty	1547
Batista operation	21
CABG + valve replacement	33
Valve replacement + Batista operation	41
Aortic surgery	79
Cardiac tumor resection	47
Congenital heart disease	366

*MI* myocardial infarction, *CABG* coronary artery bypass grafting.

The operations associated with abdominal complications after CPB included reparation of congenital ventricular septal defect (1, 3.0%), correction of congenital double outlet of right ventricle and tetralogy of Fallot (6, 18.2%), modified Fontan procedures and total cavopulmonary connections (3, 9.1%), coronary bypass grafting (3, 9.1%), valve replacement (9, 27.3%), aortic aneurysm replacement (3, 9.1%), Batista operation (1, 3%) and combined surgery (coronary bypass grafting + valve replacement, and valve replacement + Batista operation) (7, 21.2%). The mean aortic cross-clamping time in this group was 74.3 min (21-120 min) and the mean cardiopulmonary bypass time was 115 min (37-210 min).

The most common events in abdominal complications were paralytic ileus (11, 33.3%), followed by gastrointestinal bleeding (9, 27.3%), gastroduodenal ulcer with perforation (2, 6.1%), acute calculus cholecystitis(2, 6.1%), acute acalculus cholecystitis(3, 9.1%), hepatic dysfunction (4, 12.1%), and ischemia bowel diseases(2, 6.1%). Most of the abdominal complications occurred late in the postoperative period ranging from 2 to 21 days(mean 11.8 days postoperative). The incidence and the mortality of various abdominal complications are reviewed in Table 2.

**Table 2:** The incidence and the mortality of various abdominal complications

**Complications**	**Patients**	**Incidence (%)**	**Laparotomies**	**Deaths**	**Mortality (%)**
Paralytic ileus	11	33.3	0	0	0
Gastrointestinal bleeding	9	27.3	1	1	11.1
Gastroduodenal perforation	2	6.1	2	0	0
Calculus cholecystitis	2	6.1	2	0	0
Acalculus cholecystitis	3	9.1	0	0	0
Hepatic dysfunction	4	12.1	0	2	50
Ischemic bowel disease	2	6.1	2	2	100
TOTAL	33	1.4	7	5	15.2

Of these 33 patients, conservative treatments were submitted to 26 (78.8%) of them and 23 (88.5%) recovered. One patient died from gastrointestinal massive haemorrhage, and 2 died from hepatic dysfunction combined with multiple organ failure. A total of 7 patients (21.2%) had to undergo subsequent abdominal exploration. One case of duodenum bleeding, 2 of acute calculus cholecystitis and 2 of perforation with gastric ulcer were successfully surgically treated without death. Two patients with ischemic bowel disease died in spite of laparotomy. One of them was due to less ability to tolerant of the procedure and the other one was due to postoperative sepsis and circulatory failure.

In this series, 5 (15.2%) patients with abdominal complications died in all, which was significantly higher than the overall mortality (2.7%). Ischemic bowel disease and hepatic dysfunction mainly contributed to the deaths (4/5, 80%).

Some of the risk factors of abdominal complications associated with CPB are presented in Table 3. Four of 9 (44.4%) patients with postoperative gastrointestinal bleeding had a positive history of peptic ulcer. Patients who had developed abdominal complications tended to be elders. The incidence in the elders (≥75 years) is (4/74, 5.4%), which is significantly higher than those younger patients (29/2275, 1.3%, P < 0.01). Patients with unstable cardiac function or NYHA class IV were more likely to develop abdominal troubles (11/59, 18.6% vs 22/2290, 1.0%; P < 0.001). Preoperative support by IABP had been employed in 6 patients in our series, and 3 of them (50%) suffered from the complications. In the patients with abdominal complications, the operations were often much more complicated and the CPB time was significantly longer than the others (115 ± 47 min vs 69 ± 29 min). Furthermore, LCO correlated with the higher incidence of abdominal complications (16/282, 5.7% vs 17/2067, 0.8%; P < 0.001). Prolonged mechanical ventilatory support over 48 h was also associated with an increased risk (21/458, 4.6% vs 12/1891, 0.6%; P < 0.001).

**Table 3:** Risk factors of abdominal complications

	**Patients**	**Patients with abdominal complications**
Age		
≥75	74	4
<75	2275	29
Heart function		
NYHA class IV	59	11
NYHA class ≤ III	2290	22
History of peptic ulcer		
Positive	9	4
Nagetive	2340	29
Postoperative cardiac output		
Low cardiac output	282	16
Normal cardiac output	2067	17
Ventilation duration		
≥48 h	458	21
<48 h	1891	12

## Discussion

Abdominal complications associated with CPB remain a serious problem and challenge for the surgeon. These complications are often severe and carry a high mortality, resulting in a prolonged hospitalization and increased cost. The recent improvements in surgical technique and perioperative care may have been offset by the increasing complexity of cardiac surgery with older and sicker patients being operated on, as well as the large number of reoperations being done [2,3]. This makes the reported incidence of these complications varies from 0.41% to 3.7% in the last 2 decades [4]. In our department, the incidence (1.4%) was consistent with other related studies and the mortality (15.2%) was at the low horizon of these reports (13.9-63%).

A growing consensus suggests that visceral hypoperfusion during and after cardiovascular operations with CPB seems to be the common cause for abdominal complications [3]. It injures the mucosa, damages the organ, initiates the vicious circle, and serves as a trigger for the development of multiple organ failure which contributes to the high mortality from these complications [5,6]. The gastrointestinal tract, the motor of systemic inflammation, is particularly prone to be damaged from hypoperfusion and inflammatory insults [7]. Most of the patients who had a long history of congestive heart failure with poor cardiac performance have fewer abilities to tolerate or compensate for ischemia and anoxemia. Transient episodes of hypotension and indispensable hemodilution during CPB result in shunting blood away from the splanchnic area towards areas of higher priority, for example, brain. These factors induce the hepatic arterial, portal vein and gut mucosal microcirculation flow reduced [8]. CPB activates the inflammatory cells and stimulate these cells to release cytokines, which promote systemic fluid sequestration and splanchnic edema formation, and contribute to bowel microvascular barrier injury which induces the bacterial invasion and following endotoxin transfusion [9,10]. Using of vasoconstricting agents is known to worsen peripheral tissue perfusion. Taking drugs such as muscle relaxants, analgesics, and sedatives can cause enteral hypomobility. Routine administration of digitalis also induces mesenteric hypoperfusion.

Identification of risk factors for the development of abdominal complications after CPB has remained largely speculative. According to both our results and earlier studies, clinical risk factors seem to associate with following factors, advanced ages, history of peptic ulcer disease, cardiac function insufficient, the use of IABP support, prolonged CPB time, postoperative LCO and respiration dysfunction with prolonged ventilatory support [11]. Although for each complication there may be individual risk factors, as a general principle, those factors that lead to reduced peripheral blood delivery and tissue oxygenation increase the incidence significantly. The perioperative LCO syndrome is of great importance in the initiation of an incipient abdominal complication [12].

Abdominal complications after cardiovascular surgery are life-threatening. They are often parts of several postoperative events that lead to or consequences of LCO, respiratory failure, renal failure, or central nervous system deficits, ultimately result in death. As the clinical signs may be misleading and the complementary investigations may be misinterpreted, the early diagnosis is difficult in the immediate postoperative period and the patients are often critically severe at the time when the complications were recognized [13]. Managements of these serious, complicated and interwoven problems are always challenging. There-after higher mortality is inevitable. Therefore, the possibility of these critical conditions must be kept in mind, especially in patients with one or more predisposing factors, to allow early detection and treatment at a still curable stage. From a practical standpoint, patients with abdominal pain or distension should be suspected and closely monitored and the usual diagnostic measures should be undertaken appropriately.

Initial treatment is usually conservative, but when it fails, prompt surgical intervention is obligatory. The importance of early exploration cannot be overemphasized and a low threshold to early laparotomy is strongly recommended. Indeed, effective and timely operation may be life-saving in patients who are poorly able to compensate the severe haemodynamic disturbances of a refractory abdominal complication, such as major bleeding or sepsis. Experience suggests that reluctance and hesitation because the patient “had recent cardiovascular surgery” have no place if improved outcome is truly sought [14]. Placement of the abdominal incision requires special attention. The lower end of the sternal incision should be protected from contact with the abdominal incision to prevent contamination and reduce the risk of sternal infection and mediastinitis. Prophylaxis against bacterial endocarditis should be instituted, especially in patients with prosthetic vessel or valve(s). Patients who are anticoagulated require judicious reversal and reinstutition of anticoagulation after abdominal surgery.

Recently research showed that pulsatile flow during CPB may ameliorate intraoperative hypoperfusion, maintain splanchnic capillary perfusion better, and decrease the ischaemic visceral injury by mimicking the native circulation pattern. Vigorous gastric acid reduction may decrease the risk of these complications [15]. Patients in higher risk should have vigorous monitoring of gastric pH, thus prophylactic H_2_-receptor antagonists or proton pump inhibitors should routinely be given in the postoperative period. The optimisation of cardiac pump function and peripheral tissue oxygenation using preoperative IABP in patients with low CO more liberally and, possibly, infusion of energy-rich substrates may reduce the incidence of postoperative low flow states and consequently the risk of abdominal complications.

## Conclusions

In conclusion, abdominal complications after cardiovascular surgery with CPB have a low incidence but a higher mortality. Early detection and prompt appropriate intervention are essential for the outcome of the patients.

## Competing interests

The authors declare that they have no competing interests.

## Authors' contributions

GD and HW conducted the clinical follow-up and designed the research. BX and HJ acted in data analysis and interpretation. CL acted in manuscript writing. DL checked and revised the article. All authors read and approved the final manuscript.
